# Starch Characteristics Linked to Gluten-Free Products

**DOI:** 10.3390/foods6040029

**Published:** 2017-04-06

**Authors:** Stefan W. Horstmann, Kieran M. Lynch, Elke K. Arendt

**Affiliations:** School of Food and Nutritional Sciences, University College Cork, T12 Y337 Cork, Ireland; S.Horstmann@umail.ucc.ie (S.W.H.); Kieran.Lynch@ucc.ie (K.M.L.)

**Keywords:** gluten-free, morphology, composition, functional properties, modification, digestibility, starch

## Abstract

The increasing prevalence of coeliac disease (CD) and gluten-related disorders has led to increasing consumer demand for gluten-free products with quality characteristics similar to wheat bread. The replacement of gluten in cereal-based products remains a challenge for scientists, due to its unique role in network formation, which entraps air bubbles. When gluten is removed from a flour, starch is the main component left. Starch is used as gelling, thickening, adhesion, moisture-retention, stabilizing, film forming, texturizing and anti-staling ingredient. The extent of these properties varies depending on the starch source. The starches can additionally be modified increasing or decreasing certain properties of the starch, depending on the application. Starch plays an important role in the formulation of bakery products and has an even more important role in gluten-free products. In gluten-free products, starch is incorporated into the food formulation to improve baking characteristics such as the specific volume, colour and crumb structure and texture. This review covers a number of topics relating to starch; including; an overview of common and lesser researched starches; chemical composition; morphology; digestibility; functionality and methods of modification. The emphasis of this review is on starch and its properties with respect to the quality of gluten-free products.

## 1. Introduction

Patients diagnosed with coeliac disease (CD) make up 1% of the world population. However, the true number of people suffering from CD is expected to be higher [1]. CD is one of the most common food-induced diseases in humans, causing inflammation of the small intestine due to the uptake of gluten and gluten like proteins. Gluten-like proteins are the trigger for coeliac disease and are found in wheat, rye and barely. Gluten is unique due to its ability to form a visco-elastic network which can retain gas [2]. In bakery products, the gluten network formation is particularly important. However, the only treatment for patients with CD is the strict adherence to a gluten-free diet. Patients have to follow this diet throughout their life, since re-exposure to gluten can re-activate the disease, even after many years [3]. Elimination of gluten increases the role and importance of starch in providing structure and texture to gluten-free products. 

Starch is the primary source of stored energy in many plants including cereals, legumes, roots and tubers. It provides 70%–80% of the calories consumed by humans worldwide [4]. In addition to their nutritive value, starches are widely used as ingredients in many foods to impart textural and overall acceptability. They are used as gelling, thickening, adhesion, moisture-retention, stabilizing, film forming, texturizing and anti-staling ingredients. In gluten-free products, starch is incorporated into the food formulation to improve one or more of these properties. This is dependent on the interaction with other ingredients in the formulation and the type of food product [5,6]. Starch is obtained from a variety of plant sources. Corn, cassava, sweet potato, wheat, and potato are the major sources of food starch, while sorghum, barley, rice etc., serve as minor source of starch in different parts of the world. Native or raw starch occurs in the form of granules. The size, shape and molecular arrangement inside the granules depend on the plant species, and the genetic-environment interactions. The starch biosynthetic pathway generally results in two types of glucose polymers being formed, the linear amylose and the high branched amylopectin. In addition, other minor components of starch, such as proteins and lipids may be present. Cereal starches are usually considered gelling materials, and in baking they significantly contribute to texture and overall acceptability of cereal-based foods [5,6]. During dough preparation, starch absorbs up to 45% water, based on its own weight and is considered to act as inert filler in the continuous matrix of the dough [7]. On the other hand, Eliasson and Larsson [8] described bread dough as a bi-continuous network of protein and starch. During the bread baking process starch granules gelatinise i.e., they swell and are partially solubilised, but still maintain their granular identity [9]. Starch gelatinisation could play an important role in gluten-free formulation, due to the ability of starch to form a matrix in which gas bubbles are entrapped, increasing the gas holding capacity of the batters. For this reason, the addition of gel-forming starches such as pregelatinised starches and air cell stabilisers such as gums have been suggested as a means to provide gas occlusion as stabilizing agents [10]. Abdel-Aal [10] suggested three mechanisms through which addition of starch influences gluten-free formulations: enhancement of crumb softness, maintenance of the batter consistency during mixing and influencing starch gelatinisation during the baking process. Isolated wheat starch is often utilised in gluten-free products, but starch-based ingredients should ideally originate from raw materials that are naturally gluten-free [11]. Different starches from naturally gluten-free sources such as corn, cassava, potato and rice have been utilised in gluten-free formulations [12,13,14,15]. However, while rice starch has been used as basic ingredient in gluten-free bread, due to its lack of gluten, and easily digested carbohydrate [15], corn and tapioca starches can cause some difficulties by imparting an unusual taste to the product [16]. Recent studies [17,18] however, report for the application of purified gluten-free wheat starch, which is safe and does not harm the small-intestinal mucosa.

This literature review covers the topics of production and extraction of the main starches, their functional and chemical properties, digestibility and modifications (physical/chemical/enzymatic), with particular emphasis on the importance of such properties in the formulation of gluten-free products. Furthermore, a detailed table outlining the characteristics of common and less common starches is presented. 

## 2. Starch Production

Commercially available starches are obtained from various sources including wheat and corn (cereals), potato (tubers) and cassava (root). The worldwide production of starch is increasing. In three years (2009–2012) the starch production increased by 25% from approximately 60 million tonnes [19] to 75 million tonnes [20]. The recent increase in production is due to the demand in emerging countries, mainly China and Brazil (+10% per year), while other countries have a growth of 1%–2% per year [21]. Estimated starch production in 2015 were approximately 84 million tonnes [20]. According to the European starch Industry association (AAF) (www.aaf-eu.org) the main production areas of starch are North America (33%), China (33%), Europe (18%), South East Asia (11%) and South America (5%). North America, China and Europe make up 84% of the world production. The top 10 starch producing companies represent this geographic predominance. In 2010 these were Cargill (United States) with 8.0 million tonnes (MT); Ingredion (Westchester, IL, USA) with 5.9 MT; ADM (Decatur, IL, USA), 4.7 MT; Tate & Lyle (London, Great Britain), 3.6 MT; Roquette (Lestrem, France), 2.8 MT; Zhucheng Xingmao (Weifang, China), 2.0 MT; Global Bio-Chem (Hong Kong, China), 1.5 MT; Tereos Syral (Lille, France), 1.4 MT; COFCO (Beijing, China), 1.3 MT and Xiwang (Shandong, China), with 0.7 MT [21]. The main output of starch is for food applications. It represents 60% of the market, where confectionery and drinks represents 31% and processed foods, 29% [21].

## 3. Starch Sources

Over the past decades, a lot of research on starches in gluten-free formulations has been conducted. It is well known, that the characteristics of a starch (e.g., total starch, protein) affect the formulation differently. However, the quality of gluten-free products is still considered poor [22]. Table 1 gives information about the main compositional characteristics and the morphology of starches. Research conducted on flours such as acorn, bean and green plantain, has already shown that it is possible to enhance the structure and texture of gluten-free products [23,24,25,26]. Therefore, research on the less commonly used starches could identify new ingredients, which can be implemented in a gluten-free formulation. 

## 4. Starch Composition

### 4.1. Amylose/Amylopectin

**Figure 1:**
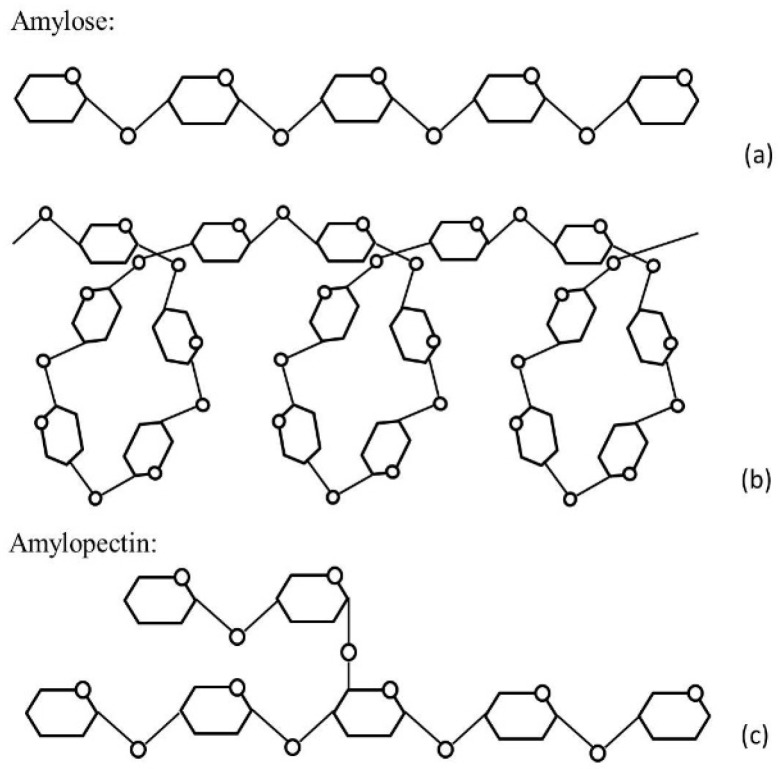
Structure of amylose ((**a**) linear (**b**) helical) and amylopectin (**c**).

Starch mainly consists of two polymers of d-glucose (Figure 1). Amylose is composed of unbranched α-1,4 linked glucose units, while amylopectin has chains of α-1,4 and also α-1,6 branching links [27,28]. Amylopectin consists of a large number of shorter chains connected together, which results in branching [29]; amylose is made up of either a single or multiple long chains, and is therefore considered as linear or a slightly branched molecule [30]. Amylose and amylopectin represent about 98%–99% of the dry weight of starch; the remaining percentages are represented by small amounts of protein, lipids, minerals and phosphorus [31]. The main sources of starch such as corn, wheat, potato, cassava, and rice contain 70%–80% amylopectin and 20%–30% amylose [32]. Nevertheless, the literature reports varying amylopectin-amylose ratios [31,33,34,35,36,37]. It has been shown, that the amylopectin–amylose ratio varies depending on the botanical origin of the starch [38] and this ratio has a significant role in the bread making process. The gelatinisation of a starch and the thermal properties of starch appear to be influenced by the amylopectin-amylose ratio as well as the amylopectin architecture [34]. Amylose and amylopectin both have an effect on the dough rheology and therefore on the structure of baked bread [33]. Furthermore, Whistler and Johnson [39] reported that the amylose content influences the nutritional and technological properties, such as susceptibility to enzymatic hydrolysis, and gelling and pasting behaviour.

### 4.2. Damaged Starch

Damaged starch is the part of starch, which is mechanically disrupted during the process of extracting and refining starch. The damaged starch content ranges from approximately 0.5% to 7.5% [33,74,75]. The level of the starch damage depends on the starch source, as well on the conditions and technique applied during the milling process. The texture of damaged starch changes the starch granule structure and therefore influences rheological and functional properties in a starch based system [44,76,77,78]. Starch damage also facilitates the swelling of the granules, due to the destruction of the structure, which prevents the swelling of the granules in water [38]. Therefore, damaged starch is considered to be more hydroscopic than native starch; as a result, damaged starch adsorbs more water than native starch [79]. Moreover, it is less resistant to enzyme activity and can be more easily degraded than intact starch granules [80]. Damaged starch also plays an important role during the fermentation process due to its susceptibility to enzymatic breakdown. The amylases present in starch generate maltose, which can be directly used for the production of carbon dioxide by the yeast, which in turn gives a rise to the dough [32]. 

### 4.3. Starch Enzymes

**Figure 2:**
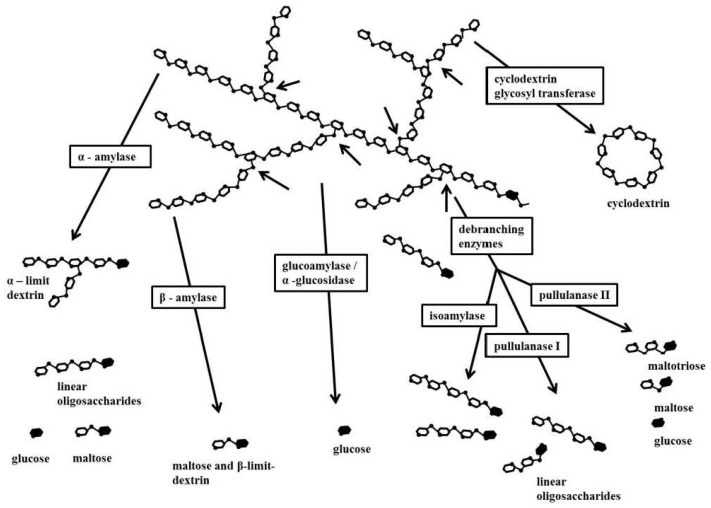
Enzymatic degradation of amylopectin. Reducing d-glucose residue (black filled); non-reducing d-glucose residue (white filled). Arrows indicate the 1,6-branch points in the starch molecule. Adapted from Antranikian [83].

Amylases are important enzymes, which can be detected in different starch sources [80]. They can be subdivided into α-amylase and β-amylase [81]. α-amylase is an endogenous-acting (endo) amylase, which is able to cleave α-1,4-glycosidic bonds, present in the inner part of the amylose and amylopectin chains (Figure 2). In contrast, β-amylase is an exogenous-acting (exo) amylase, which cleaves the exclusively α-1,4-glycosidic bonds from the non-reducing end of the chain. β-amylase acts on the external glucose residues of amylose or amylopectin, producing maltose [80]. Amylases can have a big influence on baked breads, due to the generation of reducing sugars (maltose), which can be transformed by the added yeast into carbon dioxide and alcohol [80]. A further beneficial effect of the addition of amylases is the retardation of retrogradation of amylose and, hence, decrease in the rate of staling of baked breads [82]. This could be of particular interest for gluten-free breads, as these products are based on refined starches and therefore stale faster than conventional breads. 

### 4.4. Lipids

**Figure 3:**
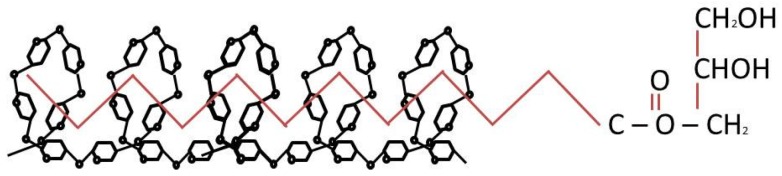
Lipid-amylose complex.

Lipids are present in commercial starch, at quantities of less than 1% [84]. These small amounts are found in the starch granules and form complexes with amylose (Figure 3). Based on the structure of the lipids, it is possible for lipids to align in the core of the amylose helix [85]. Hence, the lipids content in native starch usually correlates well with the amylose content [34]. Nevertheless, there are also surface active lipids (mainly triglycerides), which are bound by ionic or hydrogen bonds to the hydroxyl groups of the starch [86]. The lipid content of a starch is dependent on various factors, such as source, polysaccharide composition, physical structure of the grain endosperm and the amylose content [31,34]. Despite representing a minor component in starch lipids can alter the properties of starch significantly [84]. Jane [32] stated that the presence of lipids in starches increases the gelatinisation temperature, retarding granule swelling and preventing amylose from leaching out. It was further reported by Copeland et al. [34] that the amylose-lipid complexes retard the retrogradation process of heated starch. These interactions, leading to changes in the function of starch are of great benefit in the food industry [34]. The interaction of lipids in starch and its influence on the normal baking process has been comprehensively reviewed by Pareyt et al. [87]. 

### 4.5. Protein

Similar to the lipids, proteins form only a minor component of commercial starches [84]. The protein content is very much dependent on the source of starch as well as the extraction procedure. It has been reported by Jane et al. [88], that protein may be structurally bound to the starch granules and can therefore not be removed easily by the extraction process. 

## 5. Starch Morphology

**Figure 4:**
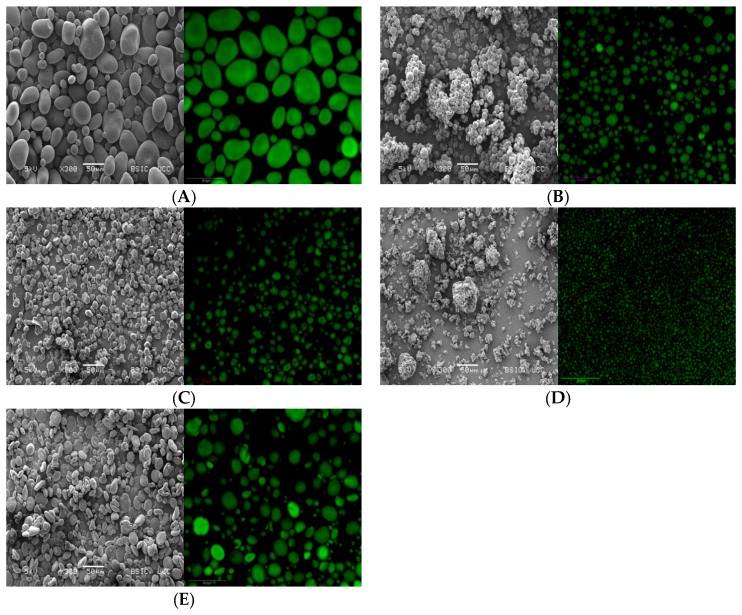
Scanning electron micrographs of (**A**) potato starch; (**B**) tapioca starch; (**C**) corn starch; (**D**) rice starch, (**E**) wheat starch. Magnification 300× (**Left**). (**Right**) Confocal laser scanning micrographs (magnification 400×).

The morphology of starch granules can be analysed by using scanning electron microscopy (SEM) or confocal laser scanning microscopy (CLSM). The shape and size of starch granules depends on the origin of starch [31]. Figure 4 shows micrographs of wheat, potato, tapioca, corn and rice starches. As shown, wheat starch has big (A) and small (B) granules ranging in size from10–35 µm and 2–10 µm, respectively. The A granules of wheat starch have a non-uniform, lenticular or disk-like shape, while the B granules are spherical or ellipsoidal granules. Potato starch also has big and small granules. The size of these granules range varies from 10–87 µm and 4–10 µm, respectively. They show a smooth surface and a round, oval shape. Furthermore, growth rings can be seen in the potato starch when observed under CLSM [33]. Tapioca starch has agglomerated starch granules with a size of 7–25 µm. The granules have both a polygonal shape and a round shape with a plain surface [89]. In comparison to tapioca starch, corn starch granules are similar in size (3–21 µm), but corn starch is only made up of polygonal shaped granules, which are not agglomerated [33,90,91]. Rice starch has granules which are agglomerated like tapioca starch granules. The polygonal shape of the granules is similar to the shape of granules in corn starch [92]. Factors which have an influence on the rheology, functional and structural properties of starch-based foods are the different granule sizes, and their shape [33]. 

## 6. Starch Digestibility

The digestion of starch is an important process with respect to dietary requirements [93]. Factors which influence the digestibility of starch are the compositional and morphological properties and the physical access of enzymes to the starch [94,95]. Starches are grouped according to their digestibly, as follows: rapidly digestible starch (RDS), slowly digestible starch (SDS) and resistant starch (RS) [96]. SDS do not increase the blood glucose level compared to RDS [97]. The blood glucose response to food is classified by the concept of the glycaemic index (GI) [98]. Benefits of SDS are a distinct hormonal and metabolic profile. Furthermore, a lower GI is linked with a reduced risk of diabetes and cardiovascular diseases [99]. Differences in starch characteristics and extraction processes have an influence on its digestibility as shown in Table 2 and Table 3, respectively. Starch digestibility plays an important role in gluten-free products, as they rely on starch as a main ingredient. Based on this, many studies have been carried out on the digestibility of gluten-free products [47,94,100,101,102,103,104,105,106]. Table 2 shows intrinsic factors influencing starch digestibility. One of these factors is the amylose/amylopectin ratio. As described in 4.1, amylose has a denser molecular structure than amylopectin. This results in amylopectin having a greater surface area, which makes it more susceptible to amylolytic attacks. This susceptibility results in faster digestion to amylose. This indicates that starch with a high amylopectin content is digested more rapidly than starch with a lower content. The lower digestibility of amylose is due to the glucose chains, which are more bound to each other by hydrogen bonds [94].This makes amylose less available to amylolytic attacks. However, as stated above other factors influence the digestibility. Minor components of starch, such as protein and fat, can form complexes with amylose and affect the enzymatic susceptibility [94]. 

As presented in Table 3, many treatments cause an increase in RDS. Most of these processes promote hydrolysis of amylose and amylopectin by gelatinisation, in excess water. In the case of baking, for example, the decreased amount of SDS is explained by reduced gelatinisation of starch, due to the lack of accessible water. In general, gelatinised starch is more easily digested, while retrograded and recrystallized starch is less digestible. Hence, cooled products after gelatinisation have reduced RDS, but increased SDS and RS content. To produce gluten-free products with decreased digestibility, modified starches with a higher amylose content could be employed. However, a higher amylose content will also ultimately change the process-ability and product quality parameters.

## 7. Starch Functionality

Starch plays a major role in food products with a variety of applications [128]. During the processing of food products, starch undergoes physico-functional changes, due to different heating and cooling steps. Major factors affecting the functionality include, amongst others, granule size and shape, starch crystallinity, amylose-amylopectin ratio, packing density, presence of fat, encapsulated starch granules, swelling power and solubility, gelatinisation, retrogradation and rheological properties [129]. Since every starch differs with respect to these properties, the selection of starch depends on the desired food properties and its production and processing [128]. 

**Figure 5:**
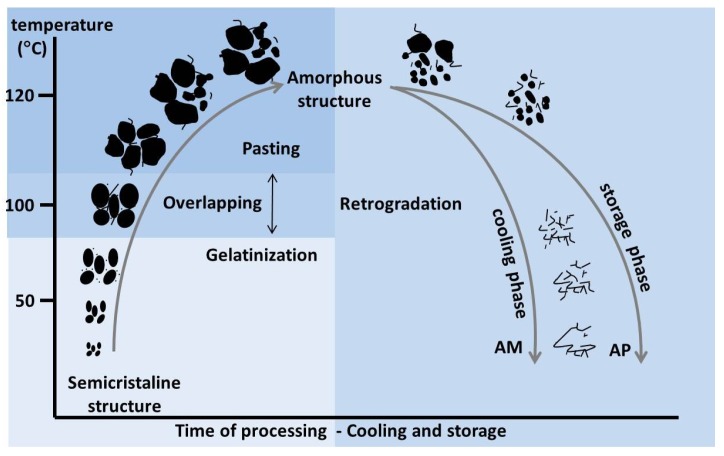
Gelatinisation, pasting and retrogradation of starch influenced by heat and time, where AM is amylose and AP amylopectin. Adapted from Schirmer et al. [129].

Native starch is insoluble in cold water; in this state starch, does not have an effect on food characteristics. However, when heat is applied to starch in excess water, the starch undergoes changes, such as gelatinisation, pasting and retrogradation (Figure 5). 

### 7.1. Gelatinisation

Native starch granules have a complex architecture. Therefore, the processing of starch granules usually involves the disruption of the structural order within the granules during heating in water [130]. This collapse or disruption of molecular order within the starch granule leads to irreversible changes in properties such as granular swelling, native crystallite melting, loss of birefringes and starch solubilisation. These changes lead to an increase in viscosity of the medium and are termed gelatinisation [131]. 

When starch is heated in water, amylose leaches from the granules [132]. The leaching of amylose and the increase of swollen granules increase the viscosity of the medium [34]. Gelatinisation is a key functional property of starch granules which determines its use in food [130]. The desired thickening property is achieved when the starch granule reaches its maximum swelling, but is not disrupted yet [129]. The temperature at which point the starch granules start to swell is referred to gelatinisation temperature. This temperature varies amongst different starch sources [41]. In general, the gelatinisation temperatures of root and tuber starches are reported to be lower than those of cereal starches [4]. 

### 7.2. Pasting

Additional heating or shear at the stage of maximal swollen granules will destruct them by disrupting hydrogen bonding between polymer chains. When this happens, a dispersion of amylose and amylopectin and granule fragments is formed, which results in loss of viscosity of the paste [129]. The point between maximal swelling and disruption leading to viscosity loss, is referred to the peak viscosity. A differentiation of pasting from gelatinization was proposed by Atwell [131]. The author defined pasting as phenomena following gelatinisation in the dissolution of starch. It involves granule swelling and eventual total disruption of the starch granule. Thomas and Atwell [4] explain that pasting cannot be separated from gelatinisation but may be described as an overlapping of these occurrences (Figure 5). Therefore, the term gelatinisation and pasting are often interchangeably used in the literature. 

### 7.3. Retrogradation

Retrogradation is the slow re-association of solubilized starch polymers to a tightly packed structure, after heating in a fluid [133]. Retrogradation of starch pastes or solutions may have the following effects: increase in viscosity, development of opacity and turbidity, formation of insoluble skins on hot pastes, precipitation of insoluble starch particles, formation of gels and syneresis of water from the paste [134]. The process of retrogradation is linked to increasing firmness or hardness of the starch [130]. In baking this process is called staling and describes the increasing firmness of bread crumb over storage time [135]. Amylose crystallises over a period of minutes to hours, while amylopectin retrogrades over hours or days [136]. Thus, the duration of the staling process is dependent on the amylose-amylopectin ratio.

Gelatinisation and pasting properties are key factors to determine the application of a starch in gluten-free breads. A simple gluten-free starch batter is a suspension of starch and yeast cells in water. The main aim during the baking process is to keep air and gas, introduced during mixing and fermentation, from escaping from the dough, and prevent starch and yeast from settling. Starch which gelatinises during the baking process increases the viscosity, which prevents settling of ungelatinised starch and the escape air and gas bubbles. The result is a set crumb structure [137]. The quick staling of gluten-free breads, as mentioned earlier, is caused by starch retrogradation of both its polymers, amylose and amylopectin [137]. In more complex gluten-free bread formulations this staling process can be retarded by the addition of ingredients such as fat, emulsifiers or other ingredients, which interact with the starch. To predict the pasting properties of starch in such gluten-free bread formulations, different methods of analysis can be used. X-ray diffraction, 13C-CP and Mas-NMR are used for the analysis of granule swelling, loss of crystallinity and of double helical order [138]. The uptake of heat is analysed using DSC (differential scanning calorimetry), and the viscosity by the RVA (Rapid Visco Analyser) [139,140]. 

## 8. State of the Art of Starch in Gluten-Free Bread Formulation and Possibilities

The formulation of gluten-free breads is a very important research area in food science. Various combinations of starch, flour, hydrocolloids, proteins and other minor ingredients have been investigated for the development of high quality gluten-free bread. Table 4 outlines recent research on gluten-free breads, including the various sources starch employed in each. It can be seen that, typically the most commonly extracted starches (potato, corn, tapioca, rice and wheat) are used in gluten-free bread. They are sometimes added as the main ingredient or as a partial substitute for gluten-free flour. This confirms that native starch plays a major role in gluten-free bread formulations. These starches can also be found in many commercial gluten-free breads (Table 5). However, gluten-free products have previously been reported, to be of lower quality than conventional breads, as reviewed recently by Rosell and Matos [141]. Despite improvements in texture and volume, consumers still rate gluten-free breads with a low acceptability. They also fear to gain weight due to higher amounts of fat, which can sometimes be twice as much as conventional breads [142]. As apparent from an examination of Table 1 and Table 4 it can be seen that there are many starches which have not yet been investigated for application in gluten-free breads. These starches could have the potential to improve the gluten-free bread quality and increase quality of gluten-free bread production. Furthermore, the use of modified starches in gluten-free bread formulations is scarce and could be of interest for gluten-free producers.

## 9. Starch Modification

Unmodified starches are used in many foods. They are used as thickener or gelling agents, for instance in gravies, custards, dressings, baked products and jelly gum candies. However, food products, made from native starches lack process ability for commercial manufacturing purposes [128]. Furthermore, the addition of ingredients to a formulation can alter the gelatinisation and pasting properties of the starch [129]. Based on this, the starch industry has developed different techniques to modify starches and improve their usefulness in food applications. 

In general, starch modification aims to produce a wide range of starches with different properties. The properties can contribute to a desirable appearance and texture, despite the above-mentioned requirements of processing and storage. Modification can make starches more process able by reducing the gelatinisation temperature or by reducing hot paste viscosity [128]. Modified starches can be mixed to achieve desired properties, such as clear pastes or freeze-thaw stability for specific products [165]. Particularly in the gluten-free baked products, modification of the starch can improve the appearance and the texture. The modifications can be split into physical and chemical modifications. This review focuses on the physical modification, since physically modified starches can be labelled as a “food starch” ingredient. In the case of chemically modified starch, it must be labelled “modified food starch” [166]. 

### 9.1. Physical Modifications

Starches can be modified, using physical methods such as: pregelatinisation and dehydration [166]. The literature on the application of modified starches in gluten-free bread formulations is scarce. However, based on the impact of pasting, gelation and retrograding properties of starch on gluten-free bread quality parameters, theses starches could compensate for lack in viscosity or lower the staling process of the bread after baking. 

The production of pregelatinised starches involves the cooking of starch, which leads to their pregelatinised state. When this state is reached, the starch is dried. Different methods of drying, such as drum drying, spray drying and extrusion cooking, are used.

Drum drying is the commonly used method by industry [128]. The method involves the use of heated, rotating metal drums. This technique leads to the formation of a starch cake in the drum. This cake is removed with a knife and then cut into the desired particle size [127]. Modified starch can swell in cold water and thicken it without the application of heat. Hence, pregelatinised starch can save time and energy, and is used to provide texture to a food [165]. 

Spray drying of starch is considered to be the best method for the production of high quality pregelatinised starch [128]. The main task in the process is the contact with high pressure steam in the chamber, which allows starch gelatinisation [167]. This treatment leads to increased functionality in cold water [130]. 

Extrusion of starch is a crude procedure, which damages the starch granules more than drum drying. It is used to lower its molecular weight and increase the solubility of the starch. Thus, a reduction in viscosity and the fragmentation of amylopectin can be achieved [130]. This leads to lower cold-water swelling powers and greater solubility [168]. The cold swelling starches modified by the above-mentioned procedures mainly find use in low-fat salad dressings, bakery fillings and dry mixes. Extruded starch finds manly applications in ready-to-eat cereals and noodles [130].

### 9.2. Other Physically Modified Starches

In addition to the pregelatinisation of starch, several other approaches have been applied to modify starch without the use of chemicals or enzyme products. Heat treatments at different moisture levels affect starch properties, such as the granule morphology, crystallinity, swelling, gelatinisation and retrogradation. These treatments are classified as heat moisture, dry heat and annealing [128]. The main aim of these treatments is to increase the pasting temperature, viscosity, process tolerance, gel temperature range, resistance to acid and shear and to reduce the swelling properties of a starch [128]. The heat moisture treatment of starches is used in sterilised soups, sauces and ambient stable products due to the increased pasting temperature [166]. 

### 9.3. Innovative Modifications of Starch

Over the last fifteen years new starch modifications, which can be applied in food systems have been introduced. 

One such modification produces superheated starch [169]. The process involves the heating of an aqueous starch suspension into the soluble state. The results of this treatment are spreadable particle gels with a creamy texture on cooling. This product is considered to be a fat-mimic in food products. The superheated starch could find application in gluten-free breads, where the fat content is higher than in conventional bread [170]. 

Modification by a multiple deep freezing and thawing method was investigated by Szymońska et al. [171]. The main procedure consisted of repeated freezing in liquid nitrogen and thawing. In the case of potato starch, it increased the specific surface area and pore size of the granules, which changed the properties of the starch. Based on the new properties it has been considered for novel applications in food technology [172].

Hydrothermal treatment of corn starch by an instantaneous controlled pressure drop (DIC) was performed by the developers, Zarguili et al. [173]. The aim was to gelatinise the starch and modify its functional properties. It is based on an abrupt transition from a high steam pressure level to a vacuum. This induces a hydro-thermal effect, which can be used to modify the functional properties of the starch for food industry needs. More recent work of the same group examined the use of this method on corn starches and wheat starch [174]. A comprehensive review by Ashogbon and Akintayo [175] describes recent trends in physical and chemical starch modifications for food and non-food applications.

## 10. Conclusions

This review covered fundamentals of starch with the aim to better understand their influence in gluten-free systems. The initial problem is that the gluten-free products which have been produced from the main starch sources until now, still lack structure and taste, compared to products containing gluten. Even though modification of starch could help improve their performance, they need to be labelled on the product, which may lead some consumers to avoid such products. This review listed a variety of starches which are grown all over the world and are yet not include in gluten-free product formulations. These starches deliver a variety of compositional and morphological properties in their native state. Thus, they should be considered in fundamental research and development of gluten-free products. Furthermore, it is noteworthy, that many different extraction methods are used even for the same source of starch. Each manufacturer of starch has its own extraction progress or at least parameters which in turn leads to a different product and results in a different chemical composition of the produced starch. These differences in composition and potential differences in physical properties have a great impact on gluten-free products and their production. Hence, a certain consistency of batch to batch quality is needed to guarantee the production of gluten-free products of consistent quality. 

## Figures and Tables

**Table 1:** Proximate composition and morphology of starches.

**Common Starch**	**Botanical Name**	**Composition**							**Granule**		**Reference**
		**Starch (%)**	**Damage Starch (%)**	**Moisture (%)**	**Amylose (%) on s.b**	**Protein (%)**	**Lipid (%)**	**Ash (%)**	**Size (µm)**	**Shape**	
**Amaranth**	*Amaranthus*	96.2	-	5.2	7.8	0.9	0.2	0.12	1.0–1.3	polygonal	[40]
**Buckwheat**	*Fagopyrum esculentum*	82.5–90.2	-	-	12.4–17.1	1.15–3.96	-	0.23–0.23	2.0–9.0	polygonal irregular, spherical	[41]
**Corn**	*Zea mays*	96.3	1.3	12.6	22.7	0.37	0.21	0.07	5.0–30.0	round, polygonal	[33]
**Waxy**		94.3	1.91	12.8	2.5	0.2	0.12	0.07	5.0–30.0	round, polygonal	[33]
**High amylose**		92.2	1.74	12.8	71	0.56	0.21	0.13	5.0–30.0	round, polygonal	[33]
**Oat**	*Avena sativa*	-	2.0–2.8	-	19.6–24.5	0.02–0.09	0.85–1.31	0.13–0.20	3.8–10.5	compound granule, polyhedral, irregular	[42]
**Potato**	*Solanum tuberosum*	93.4	0.46	14.6	20.9	0.08	0.19	0.33	15–100	oval, round	[34]
**Quinoa**	*Chenopodium quinoa*	-	-	-	10.0–21.0	-	-	-	1–2.5	compound granule, polygonal	[43]
**Rice**	*Oryza sativa*	82.4	7.4	12.5	46.4	0.04	0.7	-	3.0–8.0	compound, polygonal	[44]
**Sorghum**	*Sorghum bicolor* (L.)	81–5	-	-	14.0–23.7	0.25–0.28	-	0.10–0.14	16.0–20.0	round, polygonal	[45]
**Tapioca**	*Manihot esculenta*	95.2	0.7	13.7	36	0.03	n/a		5.0–35	compound, truncated oval	[44]
**Teff**	*Eragrostis tef*	-			28.3	0.19	0.89	0.13	1.0–2.0	compound, polyhedral	[46]
**Wheat**	*Triticum*	84.6	1.97	12.8	-	0.19	0.14	0.16	1.0–45	round, lenticular	[47]
**Uncommon Starch**		**Composition**							**Granule**		**Reference**
		**Starch (%)**	**Damage Starch (%)**	**Moisture (%)**	**Amylose (%) on s.b**	**Protein (%)**	**Lipid (%)**	**Ash (%)**	**Size (µm)**	**Shape**	
**Acorn**	*Quercus*	90–91	21%–28%	-	43–56	1.9–2.4	1.5–1.65	-	3–59	oval, irregularly round, and ovoid with diameters ranging	[48,49,50,51]
**Arracacha**	*Arracacia xanthorrhiza*	97.6–98.7	-	6.9–8.7	17.4–19.4	0.14–0.26	0.39–0.42	0.01–0.31	22–55	round and irregular shaped granules	[52,53]
**Arrowroot**	*Maranta arundinacea*	-	-	15.3	15.21	0.5	0.18	0.21	22–26	-	[54]
**Banana**	*Musa*	98.1	-	9.9	9.1–17.2	0.87–1.08		0.27–0.41	6.0–80.0	irregular in shape, elongated ovals with ridges	[55]
**Black bean**	*Phaseolus vulgaris*	-	1.5–2.0	-	27.2–29.5	0.04–0.07	0.20–0.40	0.63–0.65	7.0–30.0	round, irregular, elliptical, oval	[42]
**Breadfruit**	*Artocarpus altilis*	78.5–84.5	-	12.2–19.3	-	1.33–1.61	0.29–0.51	0.20–0.75	2.0–8.0	Small and mostly indented	[56,57]
**Cana**	*Cana edulis*	-	-	9.4–10.0	23.4–24.2	0.07–0.08	0.014–0.019	0.25–0.33	10–100	rounded and oval-shaped granules with smooth surfaces	[58,59]
**Chestnut**	*Castanea*	96.1	-	-	21.5	0.83	1.51	0.51	-	oval, irregularly round, and ovoid with diameters ranging	[60]
**Chickpea**	*Cicer arietinum* L.	94	1.6–2.1	11.9	23.3–27.2	0.57	0.1	0.05	9.0–31.0	round, irregular, elliptical, oval	[42,61]
**Cow pea**	*Vigna unguiculata* L.	93.1	-	11.5	25.8	0.49	0.15	-	16.3–22.6	morphologically irregular, oval and kidney-shaped	[61,62]
**Faba bean**	*Vicia faba* L.	90.2–90.8	-	3.0–3.6	33.7–33.9	3.88–5.37	-	0.82–1.13	6.0–25	round, elliptical, smooth surface	[61]
**Innala**	*Solenostemon rotundifolius*	-	0.1	8.9- 9.7	18.7 - 25.2	0.05 -0.07	0.25 -0.28	0.09 - 0.1	5.0–25.0	dome-shaped and hemispherical	[63]
**Kudzu**	*Pueraria hirsute Matsum*	98.6	-	12.4	22.91	0.58	-	0.52	24.08	spherical hemispherical and polygonal shaped	[64]
**Lentil**	*Lens culinaris, M.*	-	1.5–1.6	-	23.5–24.7	0.05–0.06	0.30–0.40	0.03–0.04	7.0–28.0	round, irregular, elliptical, oval	[42,65]
**Lotus**	*Nelumbo nucifera Gaertn.*	99.2	-	15.3	30.61	0.16	-	0.54	50.3	small, rounded. val shape with smooth surface	[64]
**Mung-Bean**	*V. radiate*	88.3	-	11.4	30.9–31.1	0.07–0.16	0.16–0.20	0.08	0.4–48.0	irregular, oval, round, kidney	[66,67]
**Navy bean**	*Phaseolus vulgaris*	-	1.5–1.8	-	28.2–28.6	0.07–0.08	0.3	0.60–0.65	8.0–32.0	round, irregular, elliptical, oval	[42]
**Oca**	*Oxalis tuberosa*	90.5	-	-	33	0.34	0.52	0.52	25–50	oval and elliptical shapes	[68]
**Pinto bean**	*Phaseolus vulgaris*	-	1.5–1.6	-	35.0–35.5	0.06–0.07	0.50–0.55	0.26–0.27	6.0–32.0	round, irregular, elliptical, oval	[42]
**Sago**	*Metroxylon sagu*	-	-	10.6–20.0	24–30	0.13–0.25	0.10–0.13	0.06–0.43	20–40	oval granules	[69]
**Taro**	*Colocasia esculenta*	98.9–99.0	-	7.8–7.9	27.6–35.9	0.62–0.69	0.06–0.07	0.31–0.35	1.0–12.0	polygonal, irregular shape	[70,71]
**Tania**	*Xanthosoma sagittifolium*	99.1	-	13.4	35.3	0.56	0.1	0.2	2.0–12.5	small, round, large, truncated ellipsoidal-shaped	[72]
**White yam**	*Dioscorea alata*	-	-	11.4	-	0.69	0.29	0.15	19–30	large, polyhedral and smooth	[57]
**Yam**	*Dioscorea esculenta*	-	-	8.3–11.0	20.0–31.0	0.01–0.03	0.2–0.44	0.13–0.32	3.0–45.0	polygonal/truncated oval	[73]
**Yellow pea**	*Pisum sativum*	92.3	-	11.3	31.2	0.52	0.07	-	7.0–3.2	round, elliptical, smooth surface	[61]

**Table 2:** Starch properties influencing starch digestibility.

Factors	Results	Reference
Granule size	Small granules are faster digested than bigger ones	[31,107,108]
	Small granule specific area may decrease extent of enzyme binding and result in less hydrolysis	[109]
Granule Surface	Pinholes and equatorial grooves or furrows result in faster digestion. Cereal starch faster digestible than tuber and legume starch	[110]
	Smooth surface of potato starch has high resistance to enzymatic hydrolysis	[109,111]
	Granule surface proteins and lipids block adsorption sites resulting in less enzyme binding	[112]
Composition	Native starches containing high amylose contents digest slower	[113,114,115,116]
	Amylose—lipid complexes favour restrictions towards hydrolysis	[117]

**Table 3:** Food processes altering starch digestibility.

Processes	Effect	RDS * Content	Reference
Grinding	Decrease of particle size; increase in surface area; increased hydrolysis; faster digestion	Increase	[118]
Cooking	Gelatinisation of starch; Easier available for enzymatic attack; increased hydrolysis; faster digestion	Increase	[119,120]
Extrusion Cooking	Starch loses structural integrity due to shearing and kneading, making it more susceptible towards enzymatic attacks; increased hydrolysis; faster digestion	Increase	[121,122]
Dehulling	Removal of the α-amylase inhabitants such as phytic acid, tannins, polyphenols leaving starch structure fragile and more susceptible to enzymatic degradation	Increase	[95,119]
Soaking			
Germination			
Autoclaving	Gelatinisation behaviour	Increase	[123]
Puffing	Gelatinisation behaviour	Increase	[123]
Baking	Gelatinisation behaviour	Decrease	[123]
Frying	Gelatinisation behaviour	Decrease	[123]
Roasting	Gelatinisation behaviour	Increase	[123]
Sheeting of pasta	Reduction in cohesiveness between starch and protein of dough increase amylase accessibility	Increase	[124]
Microwave cooking	penetration through microwaves increases hydrolysis	Increase	[125]
Irradiation	degradation and cross-linking of starch chains occur simultaneously during irradiation, leading to an increase in RS	Decrease	[126]
Cooling cooked food	Retrograded amylose is highly resistant to hydrolysis	Decrease	[115,116,127]

* Rapid digestible starch.

**Table 4:** Studies on the formulation of gluten-free bread including starch in the formulation.

Starch Type	Formulation	References
Corn starch	Corn starch, rice flour, cassava starch, soy flour	[142]
Cassava starch, Corn starch	Cassava starch, corn starch, rice flour, maize flour, dried milk powder, sugar, salt margarine, dried egg, baking powder, water	[143]
Corn starch	Sorghum flour, corn starch, water, salt, sugar, and dried yeast	[144]
Potato starch	Rice flour, potato starch, and skim milk powder, HPMC	[145]
Potato starch	White rice flour, potato starch, corn flour, xanthan gum, skim milk powder, soya flour, and egg powder	[146]
Corn starch, Potato starch	Corn starch, potato starch, guar gum, pectin, freeze-dried yeasts, sugar, salt, vegetable oil, water	[147]
Potato starch	HPMC, water, sorghum flour, potato starch	[148]
Potato starch, Corn starch	Potato starch, corn starch, corn meal, pectin, guar gum, xanthan gum, yeast, sugar, salt, oil, l-lysine, l-threonine, water	[149]
Corn starch	Zein, maize starch, HPMC, sugar, salt, active dry yeast	[150]
Corn starch	Corn starch, rice flour, HPMC, water, dried yeast, sunflower oil, sucrose, salt.	[151]
Cassava starch	Cassava starch, sorghum flour, water, egg white	[152]
Potato starch, Corn starch, Tapioca resistant starch, Corn resistant starch	Freeze dried yeast, oil, sucrose, salt, guar gum, pectin, potato starch, corn starch, tapioca resistant starch, corn resistant starch	[153]
Corn starch, Potato starch	Rice flour, corn flour, corn starch, potato starch, buckwheat flour, whole egg powder, whey protein, CMC, guar gum, HPMC, xanthan gum, salt, yeast, sunflower oil, water	[154]
Corn starch/Potato starch/Bean starch	Corn starch, potato starch, bean starch, premix	[24]
Corn starch/Potato starch	Corn starch, potato starch, buckwheat flour, premix	[155]
Wheat starch	Wheat starch, whey protein, locust bean gum, salt, dried active bakery yeast, d-glucose	[156]
Tapioca starch	Tapioca starch, corn flour, Salt, sugar, yeast, vegetable fat, egg, soybean flour, water	[157]
Corn starch	Corn starch, chickpea flour, pea isolate, soy flour, carob germ flour, sugar, baking powder, shortening, baker’s yeast, salt, xanthan gum, emulsifier, water	[158]
Corn starch/Potato starch	Maize starch, potato starch, guar gum, pectin, freeze dried yeasts, sucrose, salt, plant oil, water.	[159]
GF Wheat starch	Rice flour, gf wheat starch, egg albumen, fat, yeast, emulsifier mixture (DATEM, DMG), HPMC, salt, water	[23]
Pregel. Tapioca starch	Jasmine rice flour, pregel. tapioca starch, yeast, sugar, salt, shortening, water	[160]
Corn starch/Potato starch	Corn starch, potato starch, pectin, guar gum, yeasts, sugar, salt, oil, water	[25]
Corn starch/Wheat starch	Rice flour, corn starch, wheat starch, yeast, salt, oil, HPMC, white sugar	[161]
Corn Starch/Potato starch	Corn starch, potato starch, pectin, guar gum, yeast, sucrose, salt, plant oil, water	[162]
Potato starch	Rice flour, potato starch, sunflower oil, methylcellulose (MC), salt, castor sugar, dried yeast	[163]
Corn starch	Corn starch, tigernut flour, chickpea flour, shortening, sugar, baking powder, emulsifier, xanthan gum, dry yeast, salt	[164]
Potato starch, Corn starch, Wheat starch, Rice starch, Tapioca starch	Potato starch, corn starch, rice starch, gf-wheat starch, tapioca starch, water, HPMC, salt, sugar, yeast	[44]

**Table 5:** Starch and flour usage in commercial gluten-free breads (F: flour; S: starch).

Product (27)	Company	Whole Grain Maize	Maize	Rice	Potato	Tapioca	Millet	Buck-Wheat	Gluten-Free Wheat
**White Sliced Loaf**	Super Value Free From		F + S	F + S	S	S			
**Soft White Sandwich Loaf**	Genius		S	F + S	S	S			
**White Loaf**	Embrace	F + S	F + S	F + S	S	S			
**Cinnamon Raisin Loaf**	Dr. Schar		S	F					
**Multigrain Bread**	Dr. Schar		S	S	S			F	
**Deli-Style Bread**	Dr. Schar		S	F + S				F	
**Classic White bread**	Dr. Schar		S	S	S		F		
**Frozen Hearthy White bread**	Dr. Schar		S	S	S		F		
**White Sourdough Artisan Cob**	Warburtons		F + S	S	S	S			
**White Artisan Loaf**	Warburtons		F + S	S	S	S			
**White Farmhouse Loaf**	Warburtons		F + S	S	S	S			
**Seeded Farmhouse Loaf**	Warburtons		F + S	S	S	S			
**Farmhouse Loaf**	Warburtons		F + S	S	S	S			
**White Bread**	Kelkin		F + S	F	S	S			
**Multiseed Bread**	Kelkin		F + S	F	S	S			
**Sourdough Bread**	Kelkin		S		S			F	
**Multiseed Sourdough Bread**	Kelkin		S	F	S			F	
**Brown Seeded Sandwich Loaf**	Bfree		S	F	F	S		F	
**Soft White Loaf**	Bfree		S	F	F	S		F	
**Brown Bloomer Slices**	M&S		F	F	S	F			
**Brown Seeded Loaf**	M&S		F	F	S	F			
**Multigrain Farmhouse Loaf**	PureBred	F + S	S	F + S	S	F + S			
**White Farmhouse Loaf**	Purebred	F + S	S	F + S		S			
**Gluten Free Multigrain Loaf**	Has No	F + S	S	F + S	S	S			
**Gluten Free White Loaf**	Has No	F + S	S	F + S	S	S			
**Gluten Free Fibre Sliced Loaf**	Juvela								S
**Gluten Free White Sliced Loaf**	Juvela								S
